# Movable type printing method to synthesize high-entropy single-atom catalysts

**DOI:** 10.1038/s41467-022-32850-8

**Published:** 2022-08-29

**Authors:** Peng Rao, Yijie Deng, Wenjun Fan, Junming Luo, Peilin Deng, Jing Li, Yijun Shen, Xinlong Tian

**Affiliations:** 1 State Key Laboratory of Marine Resource Utilization in South China Sea, Hainan Provincial Key Lab of Fine Chemistry, School of Chemical Engineering and Technology, Hainan University, Haikou, 570228 China; 2 School of Resource Environmental and Safety Engineering, University of South China, Hengyang, 421001 China; 3 Dalian National Laboratory for Clean Energy, State Key Laboratory of Catalysis, iChEM, Dalian Institute of Chemical Physics, Chinese Academy of Sciences, Dalian, 116023 China

**Keywords:** Electrocatalysis, Catalysis, Fuel cells

## Abstract

The controllable anchoring of multiple isolated metal atoms into a single support exhibits scientific and technological opportunities, while the synthesis of catalysts with multiple single metal atoms remains a challenge and has been rarely reported. Herein, we present a general route for anchoring up to eleven metals as highly dispersed single-atom centers on porous nitride-doped carbon supports with the developed movable type printing method, and label them as high-entropy single-atom catalysts. Various high-entropy single-atom catalysts with tunable multicomponent are successfully synthesized with the same method by adjusting only the printing templates and carbonization parameters. To prove utility, quinary high-entropy single-atom catalysts (FeCoNiCuMn) is investigated as oxygen reduction reaction catalyst with much more positive activity and durability than commercial Pt/C catalyst. This work broadens the family of single-atom catalysts and opens a way to investigate highly efficient single-atom catalysts with multiple compositions.

## Abstract (web summary)

It is challenging to integrate multi-single metal atoms into one support. In this work, the authors demonstrate the production of high-entropy single-atom catalysts via a movable typing method, which enables the anchor up to eleven metals as highly dispersed single-atom active centers on the carbon support for the oxygen reduction reaction.

## Introduction

Single-atom catalysts (SACs) have attracted enormous attention due to their intrinsic advantages, such as unique geometric and electronic properties and maximum atom utilization^1–6^. It has become a booming field because of its considerable catalytic performance in various important industrial reactions, including the oxygen reduction reaction (ORR)^7–9^, oxygen evolution reaction (OER)^10–12^, hydrogen evolution reaction (HER)^13–15^, methane reforming^16–18^, methanol oxidation reaction (MOR)^19–21^, and ethanol oxidation reaction (EOR)^22–24^. In addition, numerous methods have been explored, such as high-temperature migration^25,26^, pyrolysis^27,28^, atomic layer deposition^29,30^, wet chemical^31,32^, and physical deposition^33,34^ to synthesize SACs.

In recent years, some typical SACs with non or much lower noble metals have been reported to possess impressive activity and durability that is even comparable with noble metal-based catalysts, demonstrating great potential for practical application. In addition, dual or binary single-atom catalysts (BSACs) have also been reported to exhibit better performance than their corresponding single SACs counterparts owing to the existing synergistic effects, in which the performance of the host SACs can be further modulated by the other single-metal atoms. Wang et al. reported Fe-N_4_/Pt-N_4_ isolated dual-atomic metal site SACs, and Pt-N_4_ acted as the modulator to tune the electronic structure of the Fe-N_4_ active site, efficiently promoting the ORR activity of Fe-N_4_ single-atom sites^9^. Fan et al. synthesized atomically dispersed Co_2_-N_6_ and Fe-N_4_ BSACs, and the existence of Co_2_-N_6_ efficiently tunes the electronic structure of Fe-N_4_, inducing a higher filling degree of Fe-d orbitals and optimizing the binding ability with *OH intermediates^35^. Furthermore, BSACs also play an important role in bifunctional catalysts (such as ORR and OER) owing to the multiple active centers of the BSACs. For instance, Ma et al. prepared Ni-N_4_/GHSs/Fe-N_4_ BSACs, where Ni-N_4_ and Fe-N_4_ are located on the inner and outer parts of hollow carbon supports, respectively, realizing good activity toward the ORR and OER^36^. Combined with the experimental and theoretical calculation results, Fe-N_4_ and Ni-N_4_ are confirmed as the active sites of the ORR and OER, respectively. Hu et al. reported contiguous Co-N_4_ and Fe-N_4_ BSACs anchored in N-doped carbon (FeCo-BSACs/NC), with good activity both in the ORR and OER^37^. Theoretical calculations reveal that Fe-N_4_ and Co-N_4_ act as the active centers of the ORR and OER, respectively, and the synergetic effects of Fe-N_4_ and Co-N_4_ further optimize the d-band center of the metal and improve the electrocatalytic performance.

In addition, as predicted by Beller et al. that the next breakthrough of SACs will be the preparation of bi/multimetallic SACs because of their huge potential applications in many reactions^38^. However, there have been only few works on the preparations and applications of BSACs, while multimetallic SACs, which can be expected to exhibit more attractive performance and functionalities, have rarely been reported. This is mainly attributed to the huge obstacles to the coexistence of various metals in the atomic dispersion with obviously different chemical/physical properties. Therefore, it is difficult to stabilize multiple isolated metal atoms into a single support due to the disparate coordination environment between various metal atoms and anchoring site types. Hence, developing a general and highly efficient method that can control SACs with multimetallic centers is urgently desirable but challenging.

In this work, we develop a general movable type printing method to synthesize multiple metallic SACs with up to eleven dissimilar metallic elements by transferring single metal atoms from printing templates to porous nitride-doped carbon supports (we define the SACs with more than five metal elements in a single catalyst as high-entropy single-atom catalysts (HESACs)). The single metal atoms are point-to-point transferred from the printing templates to the carbon supports under heating (see “Experimental section”), resulting in uniform mixtures of multiple elements. As expected, the prepared HESACs have typical features of uniformly dispersed single metal atoms, despite being exposed to many other metals that conventionally cause aggregation. By adjusting the amount and types of the printing templates and pyrolysis temperature, we also produce HESACs from five metals to eleven metals. Notably, as an example, the prepared quinary HESACs (FeCoNiCuMn) catalyst displays good activity and durability for both the ORR and assembled zinc-air batteries. The robust ORR performance of the quinary HESACs (FeCoNiCuMn) catalyst should be attributed to the abundant active sites and the synergistic effects between the different active sites.

## Results

### Synthesis and characterizations of HESACs

**Fig. 1:**
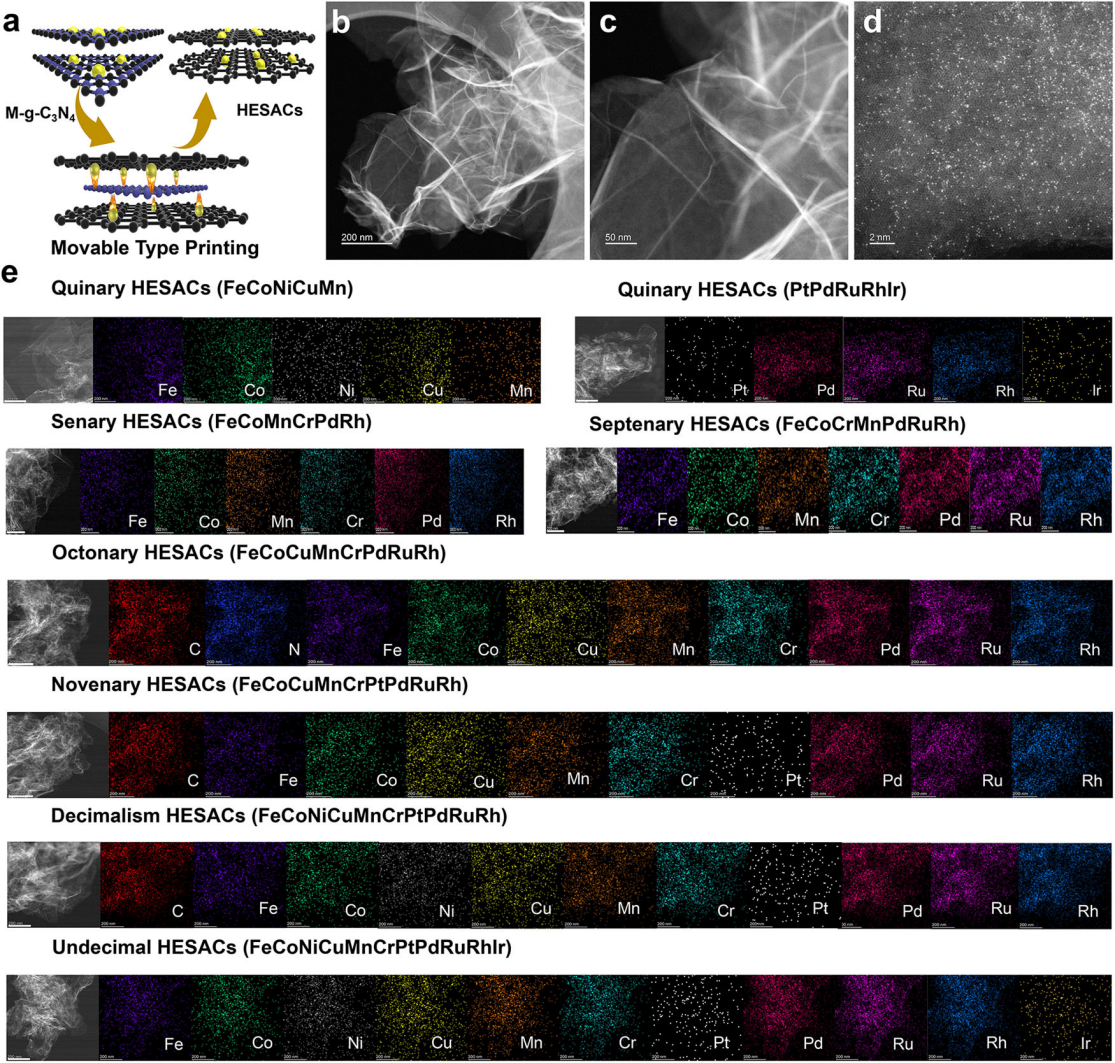
Synthesis of HESACs by movable type printing method. **a** Illustration of movable type printing method to prepare HESACs. **b**, **c** HAADF-STEM and (**d**) AC HAADF-STEM images of the quinary HESACs (FeCoNiCuMn), (**e**) STEM-EDS images of the prepared HESACs (from quinary to undecimal HESACs, scale bar is 200 nm).

The process of the movable type printing method to synthesize uniformly dispersed HESACs is shown in Fig. 1a and Supplementary Fig. 1. In brief, the metal precursors and melamine are mixed uniformly and annealed in the air to prepare the printing templates (denoted as the M-g-C_3_N_4_, Supplementary Fig. 2-3). Then, the dopamine hydrochloride was firstly coated on the prepared printing templates, and subsequently, the metal atoms are excited and stripped from M-g-C_3_N_4_ at high pyrolysis temperature, which were simultaneously captured and stabilized by the defects and nitrogen species of the carbon supports, resulting in HESACs.

High-resolution transmission electron microscopy (HRTEM) and high-angle annular dark-field scanning TEM (HAADF-STEM) images reveal that the carbon structure of prepared HESACs is highly disordered, and no metal clusters and/or nanoparticles can be observed on the carbon supports (Fig. 1b, c and Supplementary Fig. 4). In addition, XRD patterns of the prepared HESACs only show carbon peaks without any metal signals (Supplementary Fig. 5). The atomic dispersion of metal atoms is further observed using aberration-corrected HAADF-STEM (AC HAADF-STEM). The bright spots correspond to the atomically dispersed metal sites, which are homogeneously dispersed on the carbon supports (Fig. 1d and Supplementary Fig. 6). Furthermore, the energy-dispersive spectroscopy (EDS) mapping images display that the nitrogen and metals are uniform across the whole carbon supports (Fig. 1e). The results provide clear evidence for the proposed synthesis strategy to successfully synthesize from quinary to undecimal HESACs. As an example, the metal loading of quinary HESACs (FeCoNiCuMn) is characterized by inductively coupled plasma mass spectrometry (ICP–MS) measurement, and the total metal loading of the quinary HESACs (FeCoNiCuMn) is 1.47 wt.% (Supplementary Table 1).

**Fig. 2:**
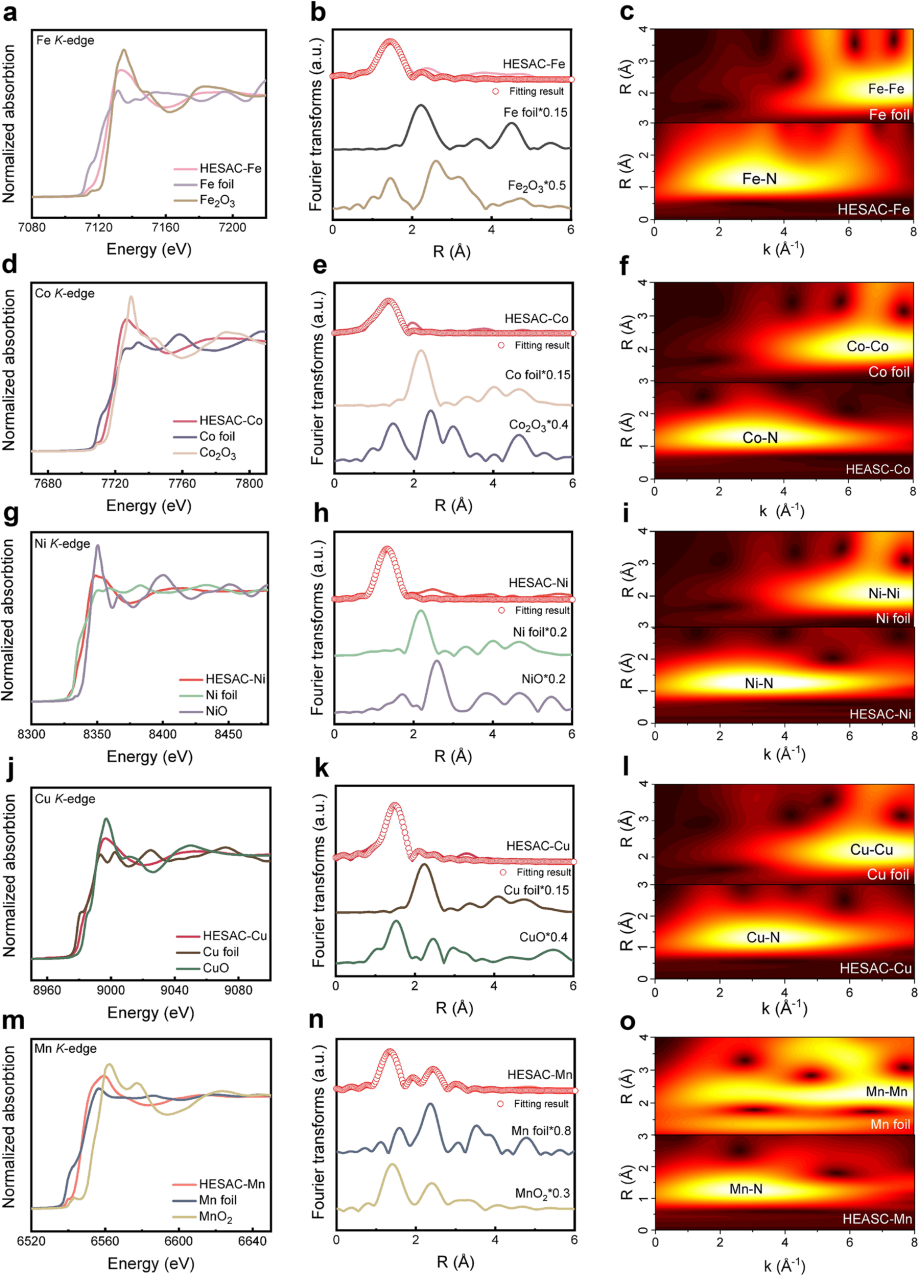
XANES and EXAFS data of prepared quinary HESACs (FeCoNiCuMn). **a, d, g, j, m** XANES spectra (**b, e, h, k, n**) of the Fe *K*-edge, Co *K*-edge, Ni *K*-edge, Cu *K*-edge and Mn *K*-edge, (**c, f, i, l, o**) WT for the EXAFS signals of the quinary HESACs (FeCoNiCuMn) and the reference samples.

To further identify the electronic structure, chemical state, and coordination environment of the prepared HESACs at the atomic level, X-ray absorption near edge structure (XANES) and extended X-ray absorption fine structure (EXAFS) are conducted on HESACs. As an illustration, the XANES and EXAFS results of the quinary HESACs (FeCoNiCuMn) and standard materials are depicted in Fig. 2. The *K*-edge absorption energies of the five metal elements are all located between the standard species of the metal foil and metal oxide, indicating that the metal species of the quinary HESACs (FeCoNiCuMn) exhibit a positive valence state (Fig. 2a, d, g, j, m). Fitting results based on the edge energies of the five metal elements and standard species (metal foil and metal oxide) indicate that the oxidation state of Fe, Co, Ni, Cu, Mn in quinary HESACs (FeCoNiCuMn) is 2.74, 1.70, 1.95, 0.34, 1.82 (Supplementary Fig. 7), consistent with the XPS results (Supplementary Fig. 8). Figure 2b, e, h, k, n displays the Fourier transformations of the EXAFS spectra (R space) of the metal and standard species, and a dominant peak corresponding to the M-N coordination shell is observed for all five elements, which provides solid evidence that the metal species of the prepared HESACs are mainly in the state of isolated metal atoms. The EXAFS fitting results and fitting parameters also confirm that the isolated metal atoms coordinated with N atoms, forming a typical M-N structure (Fig. 2b, e, h, k, n and Supplementary Table 2). Moreover, besides the domination of the M-N species, a few metallic state signals are also observed from the EXAFS data, which may be attributed to a bit metal clusters caused by the large number of metals. In addition, the atomic configuration of the quinary HESACs (FeCoNiCuMn) is further investigated by the metal (e.g., Fe, Co, Ni, Cu, Mn) *K*-edge wavelet transform (WT)-EXAFS, and compared with WT signals of the metal foil, any metal-metal coordination signal in quinary HESACs (FeCoNiCuMn) could not be observed, yet a bright and clear M-N signal was observed in all five elements of prepared quinary HESACs (FeCoNiCuMn), further identifying the isolated feature of metal species in quinary HESACs (FeCoNiCuMn) (Fig. 2c, f, i, l, o).

### Electrocatalytic ORR activity of quinary HESACs

**Fig. 3:**
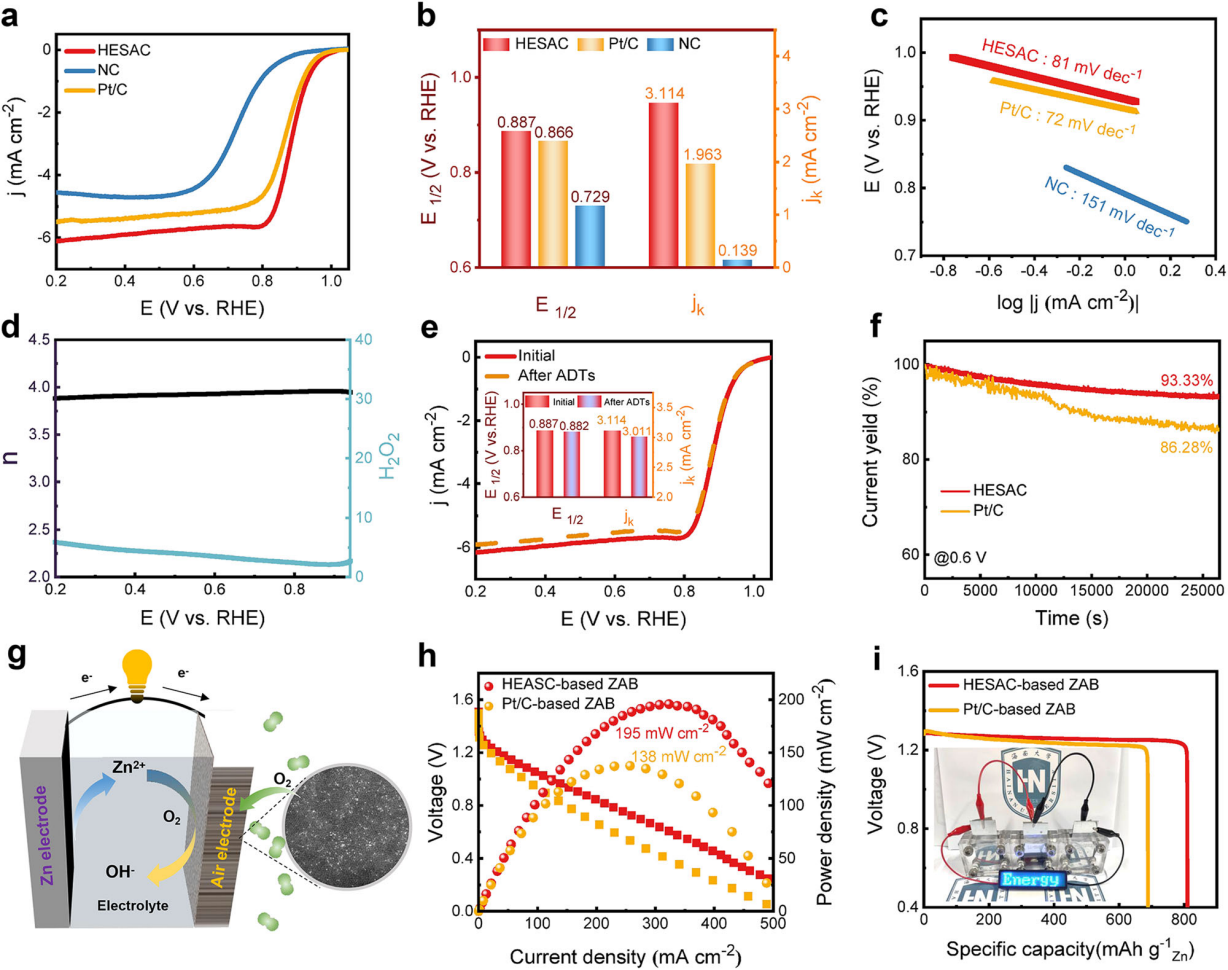
ORR performance of the prepared quinary HESACs (FeCoNiCuMn). **a** LSV curves, (**b**) comparison of E_1/2_ and j_k_, and (**c**) Tafel slopes of the HESAC, NC, and Pt/C, (**d**) RRDE and (**e**) durability data of prepared HESAC, (**f**) CA results of the HESAC and Pt/C, (**g**) schematic diagram of assembled ZAB (the inside AC HAADF-STEM image is quinary HESACs (FeCoNiCuMn)), (**h**) polarization and power curves and (**i**) specific capacity of the HESAC-based and Pt/C-based ZAB (the inside picture shows the HESAC-based ZAB in series to power the LED screen).

The movable type printing method enables diverse compositions of uniform HESACs, which exhibit great potential for a wide range of applications. As a proof of concept, we demonstrate quinary HESACs (FeCoNiCuMn) as an advanced electrocatalyst for the oxygen reduction reaction (ORR), which is the key step in many energy conversion and storage devices (such as fuel cells and metal-air batteries). Based on Brunauer-Emmett-Teller (BET) analysis, HESAC (the prepared quinary HESACs (FeCoNiCuMn) is simplified as “HESAC” in electrocatalytic measurements section) possesses a high specific area and volume of pores of 339.3 m^2^ g^−1^ and 1.8 cm^3^ g^−1^, respectively (Supplementary Fig. 9). The high specific surface area and rich pore structure of the HESAC catalyst are beneficial to promote mass transfer and expose more active sites^39–42^. In addition, the I_D_: I_G_ value of the prepared HESAC is as high as 1.076, and a high value usually indicates more defects on carbon materials, which also provide more anchored sites for single-metal atoms and active sites for the reaction process (Supplementary Fig. 10, Raman result). The ORR performance of the prepared HESAC catalyst was measured in O_2_-saturated 0.1 M KOH solution, Pt/C and nitride-doped carbon supports (also named NC) are also tested as benchmarks. The ORR activity of HESAC is more positive than that of Pt/C in terms of the onset potential (E_onset_), half-wave potential (E_1/2_), and kinetic current density (j_k_@0.9 V vs. RHE) (Fig. 3a, b, and Supplementary Table 3). The E_onset_, E_1/2_, and j_k_ of the HESAC are 0.999 V, 0.887 V, and 3.114 mA cm^−2^, which are 17 mV, 21 mV, and 1.151 mA cm^−2^ higher than those of Pt/C, respectively. To exclude the influence of the carbon supports on the ORR performance of the HESAC catalyst, an NC catalyst is prepared via a procedure same to that of the HESAC catalyst without the metal precursors. As expected, the ORR performance of pure NC is very poor (Fig. 3a, b, and Supplementary Table 3), which confirms that the good ORR performance of the HESAC should be attributed to the cooperation of the atomic metals.

The Tafel slope of the HESAC catalyst (81 mV dec^−1^) is close to that of the Pt/C (72 mV dec^−1^), indicating that the HESAC catalyst has a fast ORR kinetic process (Fig. 3c). Furthermore, the transfer number and peroxide yield of the prepared HESAC catalyst were evaluated by rotating ring-disk electrode (RRDE) measurements. The electronic transfer number is close to 4, and a low peroxide yield over the potential range of 0.20-0.95 V is obtained for the HESAC catalyst, revealing a dominant four-electron ORR process of the HESAC catalyst during the ORR process (Fig. 3d). More importantly, there is almost no activity decay of the HESAC catalyst in terms of E_1/2_ and j_k_ after accelerated durability test (ADT) (Fig. 3e). In addition, the current decay of the HESAC catalyst is only 6.67% after chronoamperometry tests for more than 25,000 s (Fig. 3f). In contrast, Pt/C displays a serious performance decline with the same durability and stability test conditions as the HESAC catalyst, indicating the robust durability and stability of the prepared HESAC catalyst (Fig. 3f, Supplementary Fig. 11, Supplementary Table 4). Moreover, it is noted that the ORR performance of the HESAC catalyst is superior to most of the reported SACs catalysts, indicating the advantages of the proposed HESACs (Supplementary Table 5).

To deeply reveal the synergistic effects of the different M-N active sites of the prepared HESAC, more controllable experiments are designed. The SAC, binary SAC (BSAC), ternary SAC (TSAC), and quaternary SAC (QSAC) are synthesized with the similar method of the prepared HESACs. For comparation, we also prepare other types binary SAC (BSAC), ternary SAC (TSAC), and quaternary SAC (QSAC) via controllable physical mixing of corresponding SAC, which are named as the BSAC-P, TSAC-P, QSAC-P, respectively. The ORR performance of the above-mentioned catalysts is shown in Supplementary Fig. 12 and Supplementary Table 6. For a fair comparison, the active centers of the catalysts are controlled in the same metal system. For example, the ORR performance of the FeCoNi-TSAC/NC only compares with the FeCo-BSAC/NC, CoNi-BSAC/NC, FeNi-BSAC/NC, Fe-SAC/NC, Co-SAC/NC, Ni-SAC/NC catalysts, without any other active centers. The ORR performance of the prepared catalysts follows the order of HESAC > QSAC > TSAC > BSAC > SAC, indicating the HESACs exhibits the best ORR activity. Moreover, we also found that the ORR performance of the BSAC, TSAC, and QSAC are much higher than those of the BSAC-P, TSAC-P, and QSAC-P, respectively, when the metal loading of the catalysts ink is controlled at similar level. Indicating besides the isolated metal active sites, there are other active contributors of the prepared BSAC, TSAC, QSAC, and HESAC, which must be attributed to the synergistic effects of the different active sites in the above catalysts. That provides one more evidence of the advance of the proposed HESACs.

To validate the potential application of the prepared HESAC catalysts, HESAC was applied as an air-cathode catalyst for the assembled ZABs (Fig. 3g). As shown in Fig. 3h, the assembled HESAC-based ZAB shows impressive ZAB performance, with a peak power density as high as 195 mW cm^−2^, which is approximately 1.4-fold higher than that of Pt/C-based ZAB (138 mW cm^−2^). The specific capacity of the HESAC-based ZAB reached 810 mAh g^−1^, which is 121 mAh g^−1^ higher than that of Pt/C-based ZAB (689 mAh g^−1^) (Fig. 3i). Moreover, the HESAC-based ZAB can stably work for over 81 h without an obvious decrease in the discharge voltage, which also reveals that the HESAC-based ZAB possesses impressive discharge stability (Supplementary Fig. 13). Furthermore, three assembled HESAC-based ZABs connected in series can power a light-emitting diode (LED) screen (Fig. 3i). All the results confirm that the prepared HESAC exhibits both good ORR performance at the rotating disk electrode (RDE) and ZAB levels, revealing high potential in practical applications of the proposed HESAC catalysts.

Furthermore, the proposed HESACs usually have more types of single-metal centers, show a great variety of active centers, and further offer multi-functional activity. Besides the ORR performance, the prepared quinary HESACs (FeCoNiCuMn) is also used in catalyzing methane oxidation and electrochemical CO_2_ reduction (ECR). The prepared quinary HESACs (FeCoNiCuMn) exhibits the best activity toward the methane oxidation, with a highest CH_3_OH prod (34.78 mmol g^−1^ h^−1^) than the prepared other SAC catalysts (Supplementary Fig. 14). Moreover, ECR of the prepared quinary HESACs (FeCoNiCuMn) was tested on the H-cell, the productions were identified as the H_2_ and CO, and the FE_CO_ is stable at about 50% (Supplementary Fig. 15). As we all know, CH_3_OH, CO, and H_2_ are the commonly used synthetic raw materials in the industry, and the prepared quinary HESACs (FeCoNiCuMn) catalyst can easily drive the reaction to produce value-added chemicals. All the data confirm that the prepared quinary HESACs (FeCoNiCuMn) is a potentially efficient catalyst for both methane oxidation and ECR, which also provide solid evidence for the presence of multiple types of active centers in the proposed HESACs. However, the activity of the prepared quinary HESACs (FeCoNiCuMn) for the methane oxidation and ECR is not as good as recently reported catalysts, which may be due to the low metal loading of the prepared quinary HESACs (FeCoNiCuMn), and our next work will focus on improving the metal loading of the HESACs, and resulting in high-efficient multi-functional catalysts.

## Discussion

In this work, we proposed the concept of HESACs, in which five or more single-metal atoms can be uniformly and stably existed on a single support. Samples from quinary to undecimal HESACs were successfully synthesized by the developed movable type printing method. As an example, the prepared quinary HESACs (FeCoNiCuMn) catalyst exhibits more positive ORR catalytic performance than that of the Pt/C catalyst and performs well in actual zinc-air battery applications. The proposal of HESACs not only enriches the types of SAC families, but also opens ideas for the development and efficient application of SACs. In addition, the proposed movable type printing method provides a good platform to study HESACs. Various metal elements with different intrinsic properties can live in harmony and exist in the form of single atoms on a single support, with the following features: (1) nonequilibrium processing, where the dispersed single metal atoms are directly point-to-point transferred to the supports by a movable type printing method, thus preventing the agglomeration of the metals; (2) controllable synthesis, where quinary to undecimal HESACs can be facilely and readily synthesized by the proposed method. The proposed movable type printing method provides a general and efficient pathway for the rational design of HESACs, which provides opportunity for the practical application of SACs in various energy-related reactions.

## Methods

### Chemicals

Melamine, FeCl_3_·6H_2_O, CrCl_3_·6H_2_O, MnCl_2_·4H_2_O_,_ NiCl_2_·6H_2_O_,_ CoCl_2_·6H_2_O, CuCl_2_·2H_2_O, H_2_PtCl_6_·6H_2_O, PdCl_2_, RuCl_3_, IrCl_3_, RhCl_3_·3H_2_O, Tris-HCl, KOH, dopamine hydrochloride, HCl, KHCO_3_, and boron nitride were purchased from Shanghai Macklin Biochemical Co., Ltd. 20% Pt/C was obtained from Johnson Matthey (JM) Corp. Nafion-117 membrane and Nafion solution (5 wt.%) were obtained from DuPont. All chemicals were used without further purification. All aqueous solutions were prepared using deionized (DI) water with a resistivity of 18.2 MΩ.

### Synthesis of printing template (M-g-C_3_N_4_)

9 g of melamine and 0.1 g of FeCl_3_·6H_2_O were added into a mixture of 120 ml of DI water and 30 ml of HCl at 110 °C under vigorous stirring, this step will stop while the solvent has evaporated. After that, the obtained solid was annealed at 550 °C for 2 h in an air atmosphere. The final sample was named as Fe-g-C_3_N_4_.

The Cr-g-C_3_N_4_, Co-g-C_3_N_4_, Ni-g-C_3_N_4_, Cu-g-C_3_N_4_, Mn-g-C_3_N_4_, Pt-g-C_3_N_4_, Pd-g-C_3_N_4_ Ru-g-C_3_N_4_, Rh-g-C_3_N_4_, and Ir-g-C_3_N_4_ were prepared via the similar method with the Fe-g-C_3_N_4_, except the mass of the metal precursor of the Cr-g-C_3_N_4_, Co-g-C_3_N_4_, Ni-g-C_3_N_4_, Cu-g-C_3_N_4_, Mn-g-C_3_N_4_, Pt-g-C_3_N_4_, Pd-g-C_3_N_4_ Ru-g-C_3_N_4_, Rh-g-C_3_N_4_, and Ir-g-C_3_N_4_ was changed to CrCl_3_·6H_2_O (0.1 g), CoCl_2_·6H_2_O (0.1 g), NiCl_2_·6H_2_O (0.1 g), CuCl_2_·2H_2_O (0.1 g), MnCl_2_·4H_2_O (0.1 g), H_2_PtCl_6_·6H_2_O (0.015 g), PdCl_2_ (0.02 g), RuCl_3_ (0.02 g), RhCl_3_·3H_2_O (0.02 g), IrCl_3_ (0.02 g), respectively.

### Synthesis of quinary HESACs (FeCoNiCuMn)

The quinary HESACs (FeCoNiCuMn) was synthesized using the desired printing templates (0.15 g of Fe-g-C_3_N_4_, 0.15 g of Co-g-C_3_N_4_, 0.1 g of Ni-g-C_3_N_4_, 0.1 g of Cu-g-C_3_N_4_, 0.1 g of Mn-g-C_3_N_4_) and 0.7 g dopamine hydrochloride dissolved in Tris-HCl. Printing templates were ultrasonically dispersed into 50 ml of Tris-HCl. After 60 min, 0.7 g of dopamine hydrochloride was added to the mixture solution under vigorous stirring. After 24 h, the reaction products were obtained by filtration and dried in a vacuum oven for 24 h. And then, the obtained solid products were annealed at 900 °C for 2 h to prepare the final product.

The synthesis of other HESACs is the same as the quinary HESACs (FeCoNiCuMn), except that the required amount of related printing templates and the pyrolysis temperature are appropriately adjusted.

### Synthesis of Fe-SAC/NC, Co-SAC/NC, Ni-SAC/NC, Cu-SAC/NC, and Mn-SAC/NC

Fe-g-C_3_N_4_ (0.15 g) was ultrasonically dispersed into 50 mL Tris-HCl solution. After 60 min, 0.7 g of dopamine hydrochloride was added to the mixture solution under vigorous stirring. After 24 h, the reaction products were obtained by filtration and dried in a vacuum oven for 24 h. And then, the obtained solid products were annealed at 900 °C for 2 h to prepare the final product, and the final product was labeled as Fe-SAC/NC.

The preparation of Co-SAC/NC, Ni-SAC/NC, Cu-SAC/NC, and Mn-SAC/NC is the same as the Fe-SAC/NC, except Co-g-C_3_N_4_ (0.15 g), Ni-g-C_3_N_4_ (0.10 g), Cu-g-C_3_N_4_ (0.10 g), and Mn-g-C_3_N_4_ (0.10 g) was used as the printing templates of Co-SAC/NC, Ni-SAC/NC, Cu-SAC/NC, and Mn-SAC/NC, respectively.

### Synthesis of FeCo-DSAC/NC, FeCu-DSAC/NC, and CoNi-DSAC/NC

Fe-g-C_3_N_4_ (0.15 g) and Co-g-C_3_N_4_ (0.15 g) were ultrasonically dispersed into 50 mL Tris-HCl solution. After 60 min, 0.7 g of dopamine hydrochloride was added to the mixture solution under vigorous stirring. After 24 h, the reaction products were obtained by filtration and dried in a vacuum oven for 24 h. And then, the obtained solid products were annealed at 900 °C for 2 h to prepare the final product, the final product was labeled as FeCo-DSAC/NC.

The preparation of FeCu-DSAC/NC and CoNi-DSAC/NC is the same as the FeCo-DSAC/NC, except Fe-g-C_3_N_4_ (0.15 g) and Cu-g-C_3_N_4_ (0.10 g), Co-g-C_3_N_4_ (0.15 g) and Ni-g-C_3_N_4_ (0.10 g) were used as the printing templates of FeCu-DSAC/NC and CoNi-DSAC/NC, respectively.

### Synthesis of FeCoMn-TSAC/NC and FeCuMn-TSAC/NC

Fe-g-C_3_N_4_ (0.15 g), Co-g-C_3_N_4_ (0.15 g), and Mn-g-C_3_N_4_ (0.10 g) were ultrasonically dispersed into 50 mL Tris-HCl solution. After 60 min, 0.7 g of dopamine hydrochloride was added to the mixture solution under vigorous stirring. After 24 h, the reaction products were obtained by filtration and dried in a vacuum oven for 24 h. And then, the obtained solid products were annealed at 900 °C for 2 h to prepare the final product, and the final product was labeled as FeCoMn-TSAC/NC.

The preparation of FeCuMn-TSAC/NC is the same as the FeCoMn-TSAC/NC, except Fe-g-C_3_N_4_ (0.15 g), Cu-g-C_3_N_4_ (0.10 g), and Mn-g-C_3_N_4_ (0.10 g) were used as the printing templates of FeCuMn-TSAC/NC.

### Synthesis of FeCoCuMn-QSAC/NC, FeCoNiCu-QSAC/NC, and FeCoNiMn-QSAC/NC

Fe-g-C_3_N_4_ (0.15 g), Co-g-C_3_N_4_ (0.15 g), Cu-g-C_3_N_4_ (0.10 g), and Mn-g-C_3_N_4_ (0.10 g) were ultrasonically dispersed into 50 mL Tris-HCl solution. After 60 min, 0.7 g of dopamine hydrochloride was added to the mixture solution under vigorous stirring. After 24 h, the reaction products were obtained by filtration and dried in a vacuum oven for 24 h. And then, the obtained solid products were annealed at 900 °C for 2 h to prepare the final product, the final product was labeled as FeCoCuMn-QSAC/NC.

The preparation of FeCoNiCu-QSAC/NC and FeCoNiMn-QSAC/NC is the same as the FeCoCuMn-QSAC/NC, except Fe-g-C_3_N_4_ (0.15 g), Co-g-C_3_N_4_ (0.15 g), Ni-g-C_3_N_4_ (0.10 g), Cu-g-C_3_N_4_ (0.10 g), and Fe-g-C_3_N_4_ (0.15 g), Co-g-C_3_N_4_ (0.15 g), Ni-g-C_3_N_4_ (0.10 g), Mn-g-C_3_N_4_ (0.10 g) were used as the printing templates of FeCoNiCu-QSAC/NC and FeCoNiMn-QSAC/NC, respectively.

### Material characterizations

The TEM and HRTEM were tested by using a Thermo Scientific Talos F200X G2 operated at 200 keV. AC HAADF-STEM was tested by aberration-corrected FEI Titan G2 60–300 field-emission TEM (FEI, USA), operated at 300 keV (αmax = ~100 mrad). XRD was conducted on an HAOYUAN powder diffractometer (DX-2700BH) operated at 60 kV and 50 mA, using a Cu-K radiation source. X-ray photoelectron spectroscopy (XPS) was performed on a Thermo Scientific K-Alpha X-ray photoelectron spectrometer employing a monochromated Al-K X-ray source (hν = 1486.6 eV). Raman spectra were recorded at ambient Horiba Scientific Raman Spectrometer using the exciting line at 532 nm of a diode Laser. The metal loading was determined by using ICP-MS (Agilent ICPMS 7700).

The X-ray absorption fine structure (XAFS) spectra were collected at 1W1B beamline of Beijing Synchrotron Radiation Facility (BSRF). The tested samples were prepared by pressing the catalyst and boron nitride. The data were collected in fluorescence mode using a Lytle detector and the corresponding reference samples were collected in transmission mode.

The acquired XAFS data were processed according to the standard procedures using the ATHENA module of Demeter software packages. To obtain the quantitative structural parameters around central atoms, least-squares curve parameter fitting was performed using the ARTEMIS module of Demeter software packages.

The following EXAFS equation was used:

1 $$\chi (k)=\mathop{\sum}\limits_{j}\frac{{N}_{j}{S}_{0}^{2}{F}_{j}(k)}{k{R}_{j}^{2}}\cdot exp[-2{k}^{2}{\sigma }_{j}^{2}]\cdot exp[\frac{-2{R}_{j}}{\lambda (k)}]\cdot sin[2k{R}_{j}+{\phi }_{j}(k)]$$

### Electrochemical measurements

In brief, the ORR performance tests were conducted on Gamry 1010E electrocatalytic station. A glass carbon electrode (GCE), a carbon rod, and a Hg/HgO was applied as working electrode, counter electrode, and reference electrode, respectively. The electrolyte was composed of the 0.1 M KOH. The catalysis ink was prepared via uniformly disperse of 5 mg catalysts, 970 μL of ethanol and 30 μL Nafion. After that, drop 7 μL of the catalysis ink onto the GCE to obtain the testing working electrode. The LSV curves were recorded at 1600 revolutions per minute (rpm)) with a scan rate of 5 mV·s^−1^ in O_2_-saturated 0.1 M KOH. The accelerated durability test (ADT) method was applied to test the durability, with a 20,000 cycles potential cycling from 0.6 to 1.0 V at 100 mV s^−1^. The stabilities of the catalyst were studied by chronoamperometry (CA) at 0.6 V vs. RHE in an O_2_-saturated electrolyte.

Rotating ring-disk electrode (RRDE) measurements of the samples was measured to study the four-electron selectivity. The Pt ring electrode was biased at 1.1 V vs. RHE. The H_2_O_2_ yield and n per oxygen molecule were calculated by the following equations:

2 $$\%{H}_{2}{O}_{2}=200\frac{{I}_{R}/N}{{I}_{D}+{I}_{R}/N}$$

3 $${{{\rm{n}}}}=4\frac{{I}_{D}}{{I}_{D}+{I}_{R}/N}$$

where I_D_ and I_R_ are the disk and ring currents, respectively. N is the ring current collection efficiency (37%).

All potentials in this work are quoted with respect to a reversible hydrogen electrode (RHE). The potentials in LSV curves in this study have subjected to iR compensation.

### Zinc-air battery (ZAB) test

The ZAB was fabricated as following: a polished Zn plate, catalysts-loaded carbon paper (1 mg cm^−2^), and a mixture solution of 6 M KOH and 0.2 M Zn(Ac)_2_ was used as the anode, cathode, and electrolyte, respectively. A Gamry 1010E electrochemical workstation was used to recorded the polarization curves. The discharge polarization curve was carried out on LANHE (CT2001A) battery testing system. The specific power density was calculated by P = j * E, in which P is power density, j is current density, E is voltage.

### Direct conversion of methane to methanol

The direct oxidation of methane was carried out in a 50 ml autoclave containing 10 ml of catalyst solution. The autoclave was sealed and purged three times with argon containing gas and the pressure was maintained at 3.0 MPa gas mixture (H_2_: 3.3%, O_2_: 6.6%, CH_4_: 1.6%, Ar: 61.7%, and He: 26.8%), the mixture was stirred at 1200 rpm and heated to 70 °C with a ramp rate of 1.5 °C min^−1^ and held for 0.5 h. At the end of the reaction, the autoclave was kept to ice water for 2 h to minimize the loss of volatile products.

### Electrochemical CO_2_ reduction (ECR) performance measurements and product analysis

Electrochemical tests were conducted on a workstation (CHI 760E). A gas-tight H-cell reactor segregated with a Nafion-117 membrane was used for the electrolysis experiments. In the cathodic chamber (100 mL), an L-type GCE and Ag/AgCl served as the working and reference electrode, respectively. A Pt foil in the anodic chamber (100 mL) served as a counter electrode. Each chamber contained 50 mL KHCO_3_ electrolyte (0.1 M, pH = 6.8). Carbon dioxide was continuously purged (30 mL min^−1^) during the ECR process. All potentials were recalculated into RHE by E_RHE_ = E_Ag/AgCl_ + 0.1989 + 0.0591 × pH according to Nernst equation unless otherwise stated.

The cathodic energy efficiency (EE) was calculated as follows:

4 $${EE}\left(100\%\right)=\frac{1.23-{E}^{0}}{1.23-E}\times {FE}$$

where E^0^, FE and E represented standard potential, faradaic efficiency and applied potential, respectively.

## Supplementary information

Supplementary Information

## Data Availability

The data that support the findings of this study are available from the manuscript and its supplementary information. Source data are provided with this paper.

## Source data

Source Data
