# Supplementary material for: Movable type printing method to synthesize high-entropy single-atom catalysts
Source: Nat Commun. 2022 Aug 29;13:5071. doi: 10.1038/s41467-022-32850-8 (PMC9424199; doi:10.1038/s41467-022-32850-8)
Supplement: Supplementary file 1 — Supplementary Information [file 41467_2022_32850_MOESM1_ESM.pdf]

Supplementary information for

Movable type printing method to synthesize high-entropy single-atom catalysts

Peng Rao[1], Yijie Deng[2], Wenjun Fan[3], Junming Luo[1], Peilin Deng[1], Jing Li[1], Yijun Shen[1], and Xinlong Tian[1,*]

[1]State Key Laboratory of Marine Resource Utilization in South China Sea, Hainan Provincial Key Lab of Fine Chemistry, School of Chemical Engineering and Technology, Hainan University, Haikou 570228, China

[2]School of Resource Environmental and Safety Engineering, University of South China, Hengyang 421001, China

[3]Dalian National Laboratory for Clean Energy, State Key Laboratory of Catalysis, iChEM, Dalian Institute of Chemical Physics, Chinese Academy of Sciences, Dalian 116023, China

***Corresponding author**: tianxl@hainanu.edu.cn (X. Tian)*

Suplmentarry

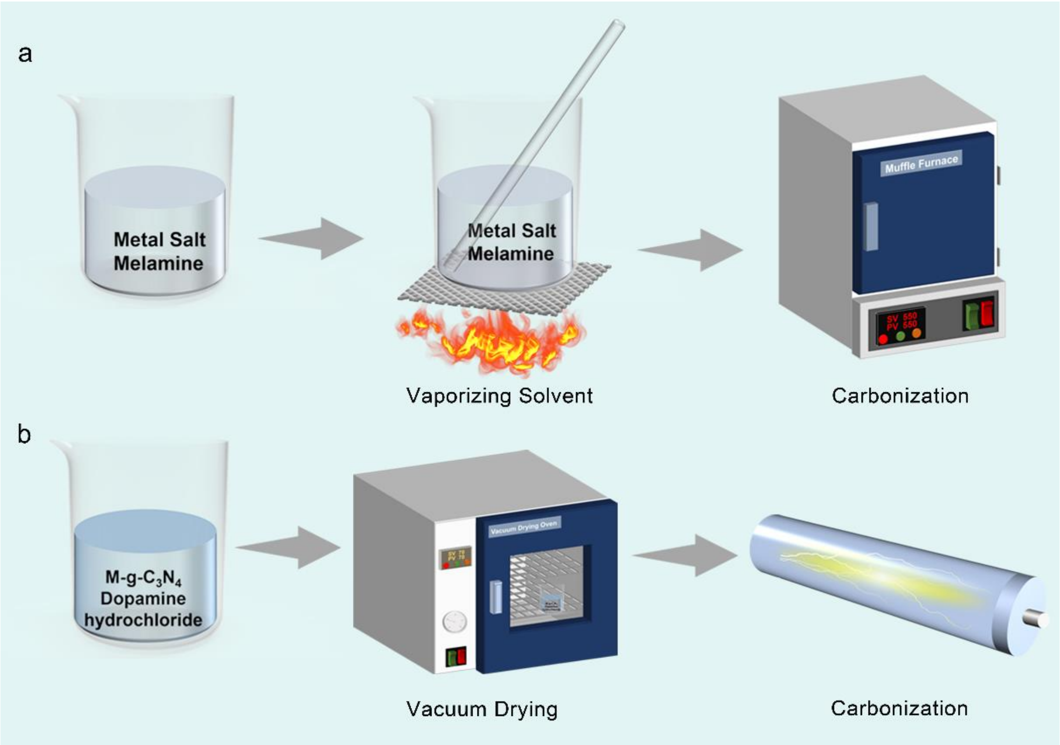

Supplementary Fig. 1 Schematic of the preparation of single-atom catalysts. **(a)** Preparation process of the M-g-C_3N_4, **(b)** Preparation process of the HESACs.

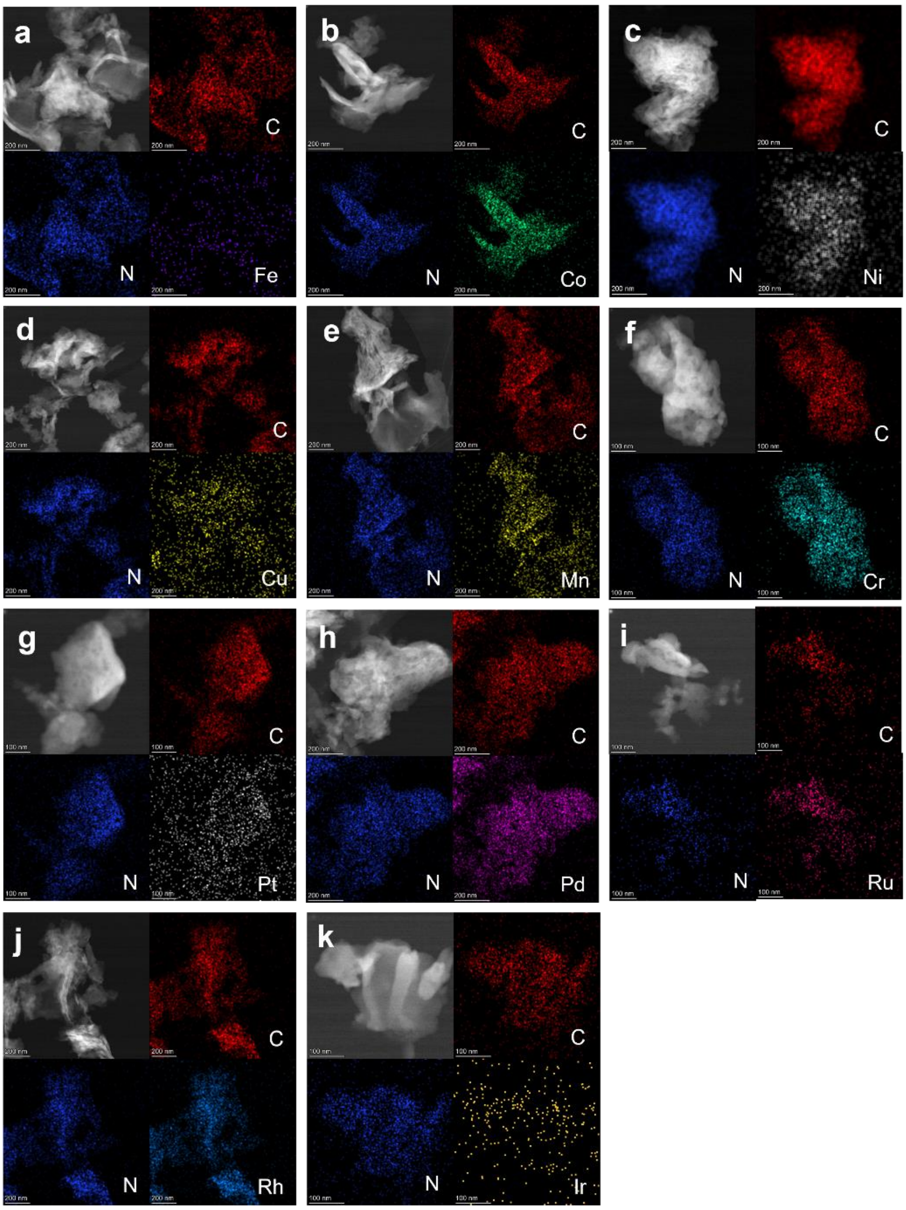

Supplementary Fig. 2 HAADF-STEM and EDS mapping images of the prepared M-g-C_3N_4. HAADF-STEM and EDS mapping images of **(a)** Fe-g-C_3N_4, **(b)** Co-g-C_3N_4, **(c)** Ni-g-C_3N_4, **(d)** Cu-g-C_3N_4, **(e)** Mn-g-C_3N_4, **(f)** Cr-g-C_3N_4, **(g)** Pt-g-C_3N_4, **(h)** Pd-g-C_3N_4, **(i)** Ru-g-C_3N_4, **(j)** Rh-g-C_3N_4, **(k)** Ir-g-C_3N_4.

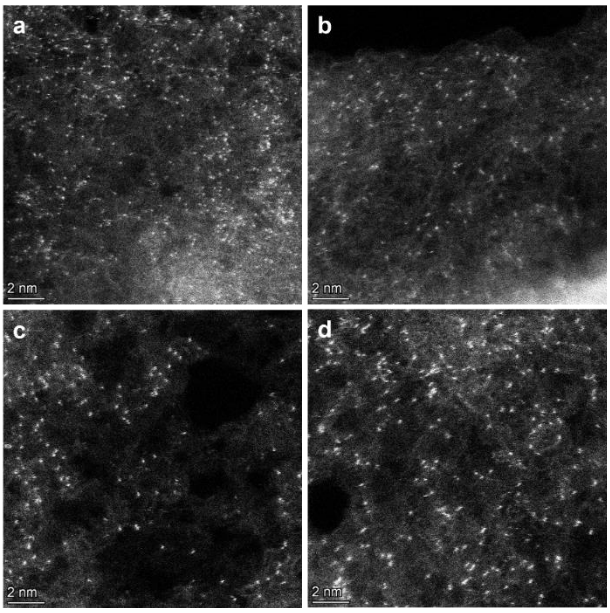

Supplementary Fig. 3 AC HAADF-STEM Images of the prepared templates. AC HAADF-STEM images of the prepared **(a)** Fe-g-C_3N_4, **(b)** Co-g-C_3N_4, **(c)** Cu-g-C_3N_4, and **(d)** Mn-g-C_3N_4.

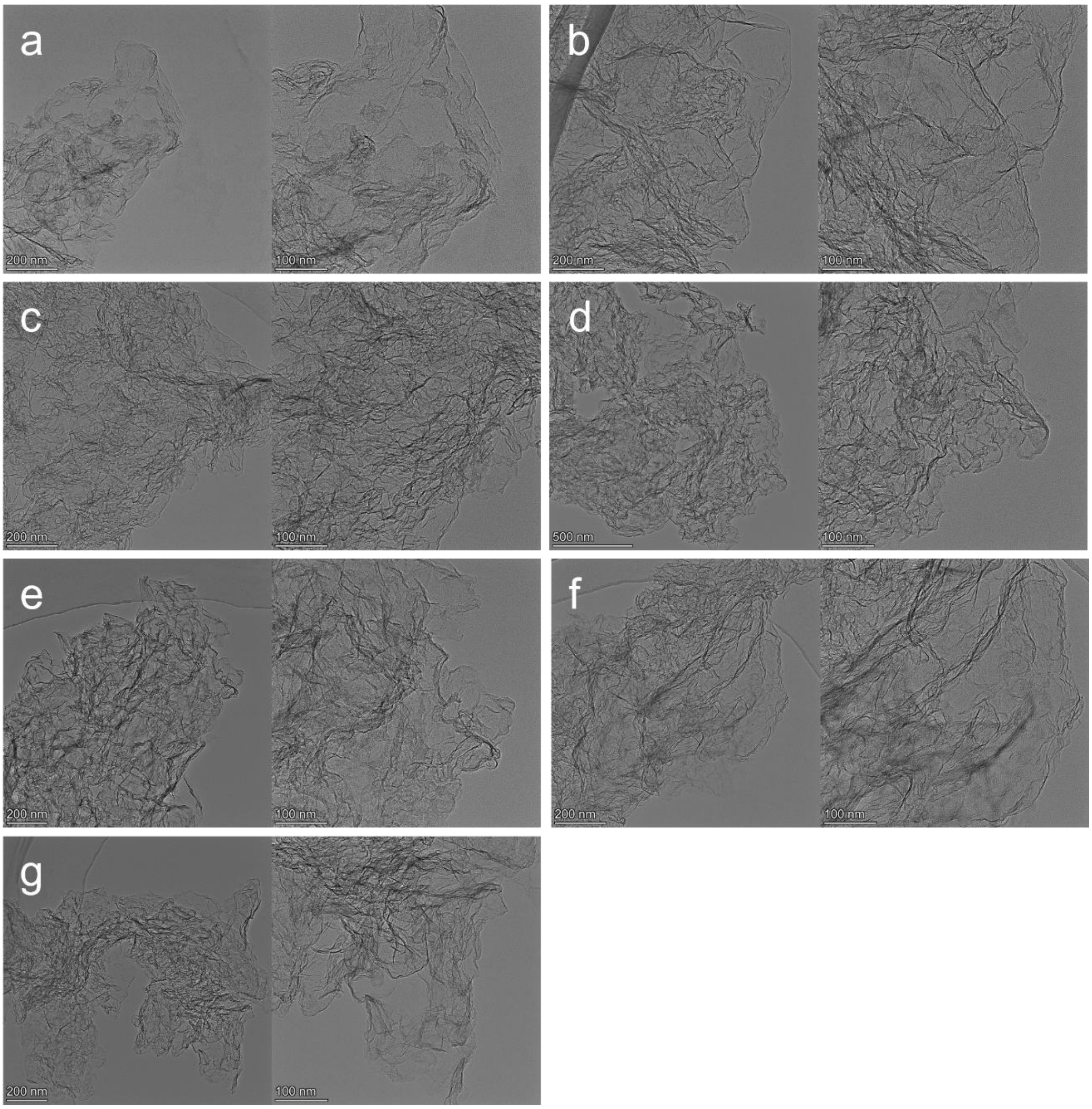

Supplementary Fig. 4 HRTEM images of the prepared HESACs. **(a)** quinary HESACs (PtPdRuRhIr), **(b)** senary HESACs (FeCoMnCrPdRh), **(c)** septenary HESACs (FeCoCrMnPdRhRu), **(d)** octonary HESACs (FeCoCuMnCrPdRuRh), **(e)** novenary HESACs (FeCoCuMnCrPtPdRuRh), **(f)** decimalism HESACs (FeCoNiCuMnCrPtPdRuRh), **(g)** undecimal HESACs (FeCoNiCuMnCrPtPdRuRhIr).

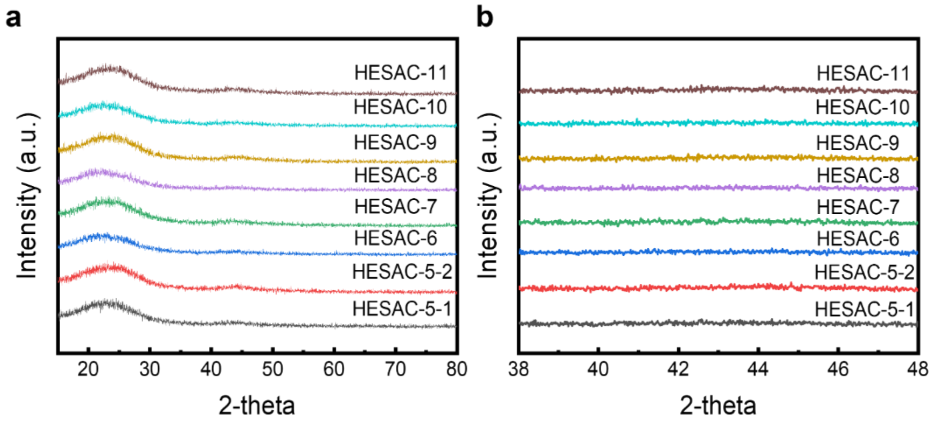

Supplementary Fig. 5 XRD patterns of the prepared catalysts. Full **(a)** and local **(b)** XRD patterns of the prepared HESACs.

HESAC-5-1: quinary HESACs (FeCoNiCuMn)

HESAC-5-2: quinary HESACs (PtPdRuRhIr)

HESAC-6: senary HESACs (FeCoMnCrPdRh)

HESAC-7: septenary HESACs (FeCoCrMnPdRhRu)

HESAC-8: octonary HESACs (FeCoCuMnCrPdRuRh)

HESAC-9: novenary HESACs (FeCoCuMnCrPtPdRuRh)

HESAC-10: decimalism HESACs (FeCoNiCuMnCrPtPdRuRh)

HESAC-11: undecimal HESACs (FeCoNiCuMnCrPtPdRuRhIr)

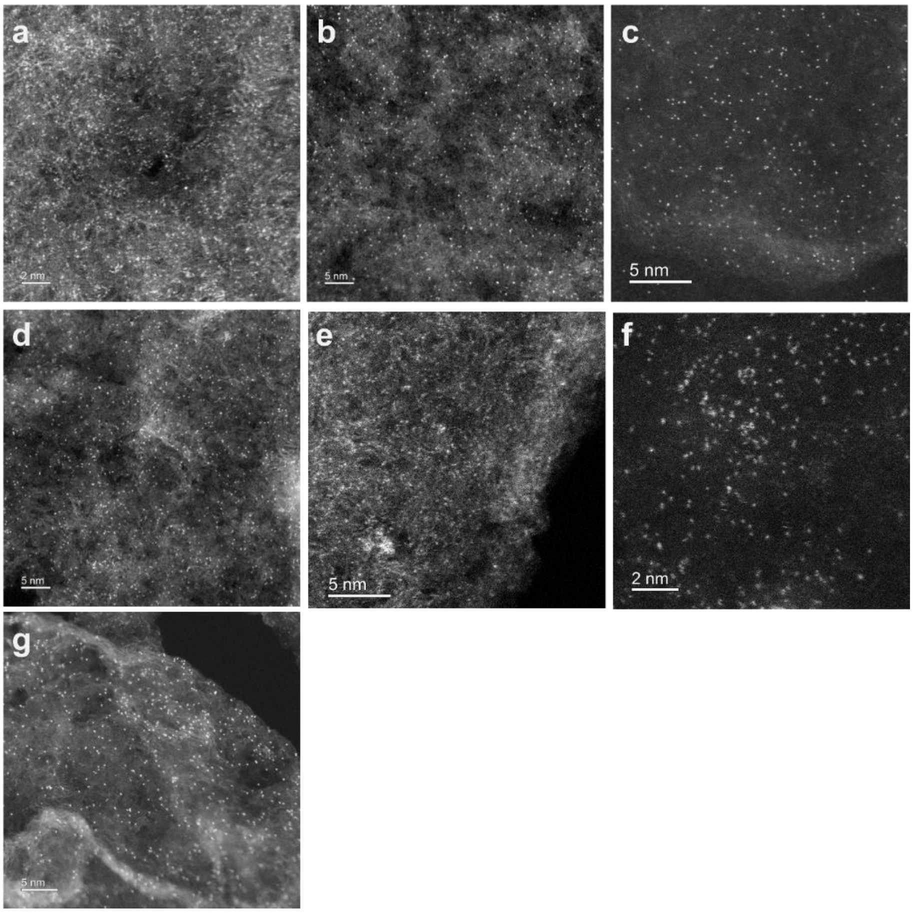

Supplementary Fig. 6 AC HAADF-STEM images of the prepared HESACs. **(a)** quinary HESACs (PtPdRuRhIr), **(b)** senary HESACs (FeCoMnCrPdRh), **(c)** septenary HESACs (FeCoCrMnPdRhRu) **(d)** octonary HESACs (FeCoCuMnCrPdRuRh), **(e)** novenary HESACs (FeCoCuMnCrPtPdRuRh), **(f)** decimalism HESACs (FeCoNiCuMnCrPtPdRuRh), **(g)** undecimal HESACs (FeCoNiCuMnCrPtPdRuRhIr).

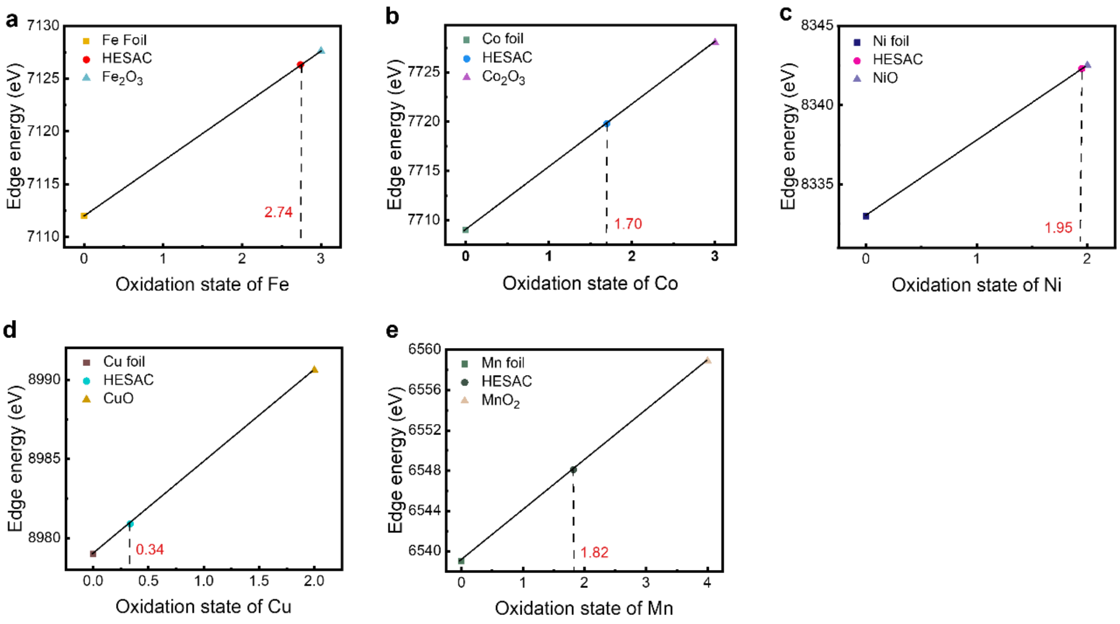

Supplementary Fig. 7 The oxidation state of the metal in quinary HESACs (FeCoNiCuMn). Oxidation state analysis of **(a)** Fe, **(b)** Co, **(c)** Ni, **(d)** Cu, and **(e)** Mn elements in prepared HESAC. The oxidation state fitting was based on edge energy.

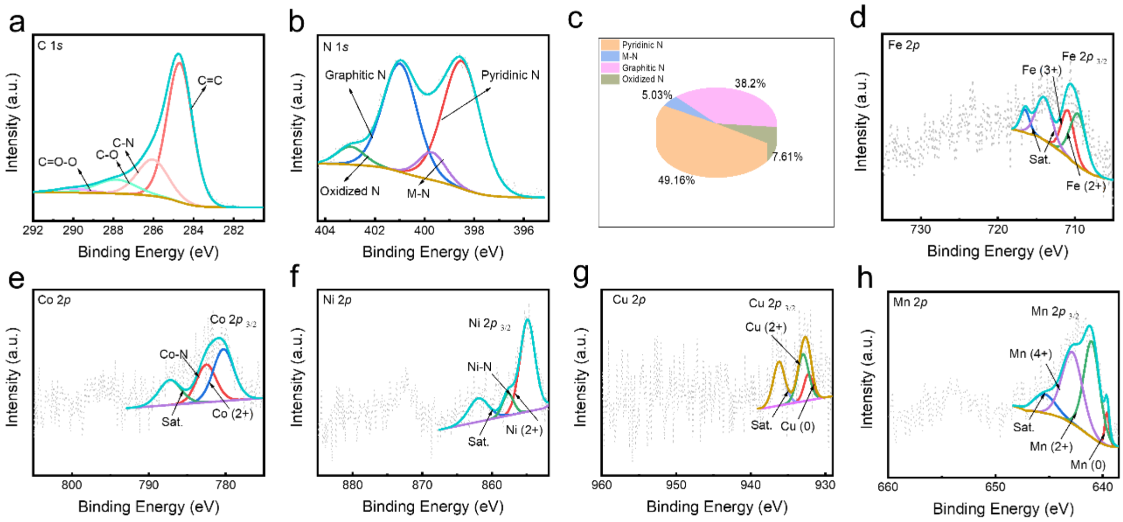

Supplementary Fig. 8 XPS results of the quinary HESACs (FeCoNiCuMn). **(a)** C 1*s* and **(b)** N 1*s* high-resolution XPS peaks, **(c)** the content of the N species, **(d)** Fe 2*p*, **(e)** Co 2*p*, **(f)** Ni 2*p*, **(g)** Cu 2*p*, **(h)** Mn 2*p* high-resolution XPS peaks of the prepared quinary HESACs (FeCoNiCuMn).

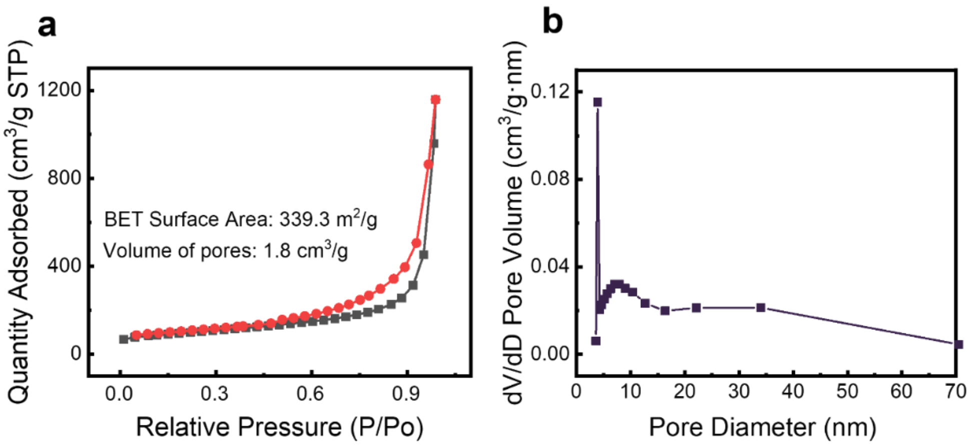

Supplementary Fig. 9 Specific surface area pore distribution. **(a)** BET result and **(b)** pore distribution and volume result of the prepared quinary HESACs (FeCoNiCuMn).

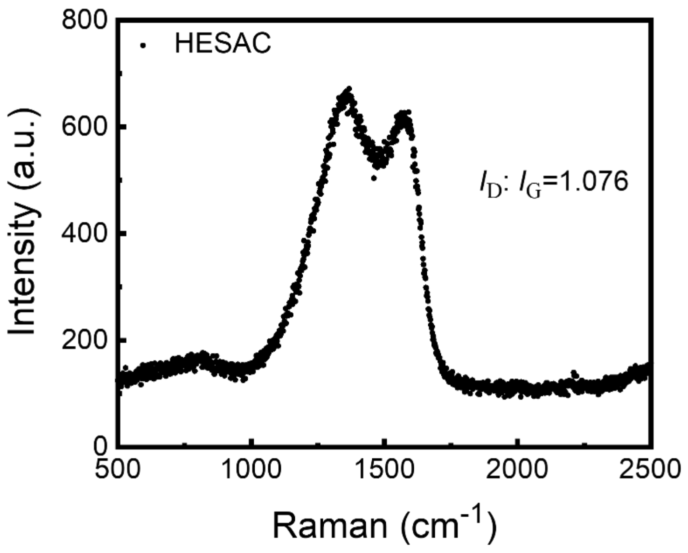

Supplementary Fig. 10 Raman characterization. Raman result of the prepared quinary HESACs (FeCoNiCuMn).

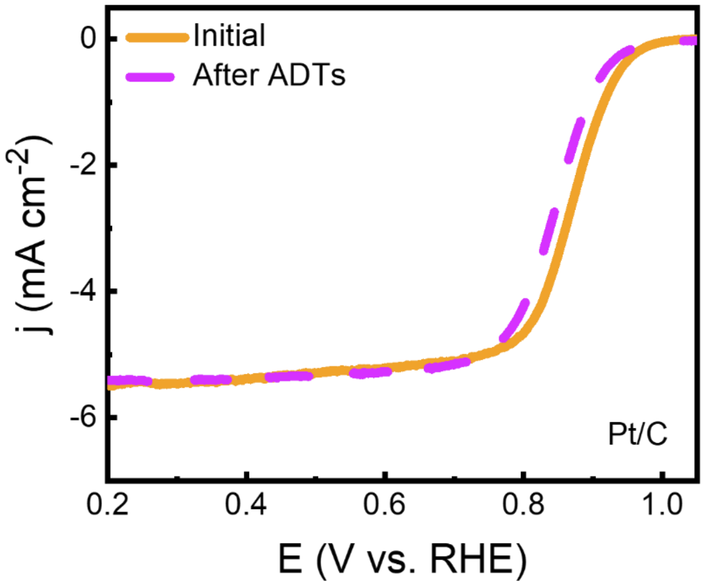

Supplementary Fig. 11 ADT results of Pt/C. LSV curves of the Pt/C before and after ADT test.

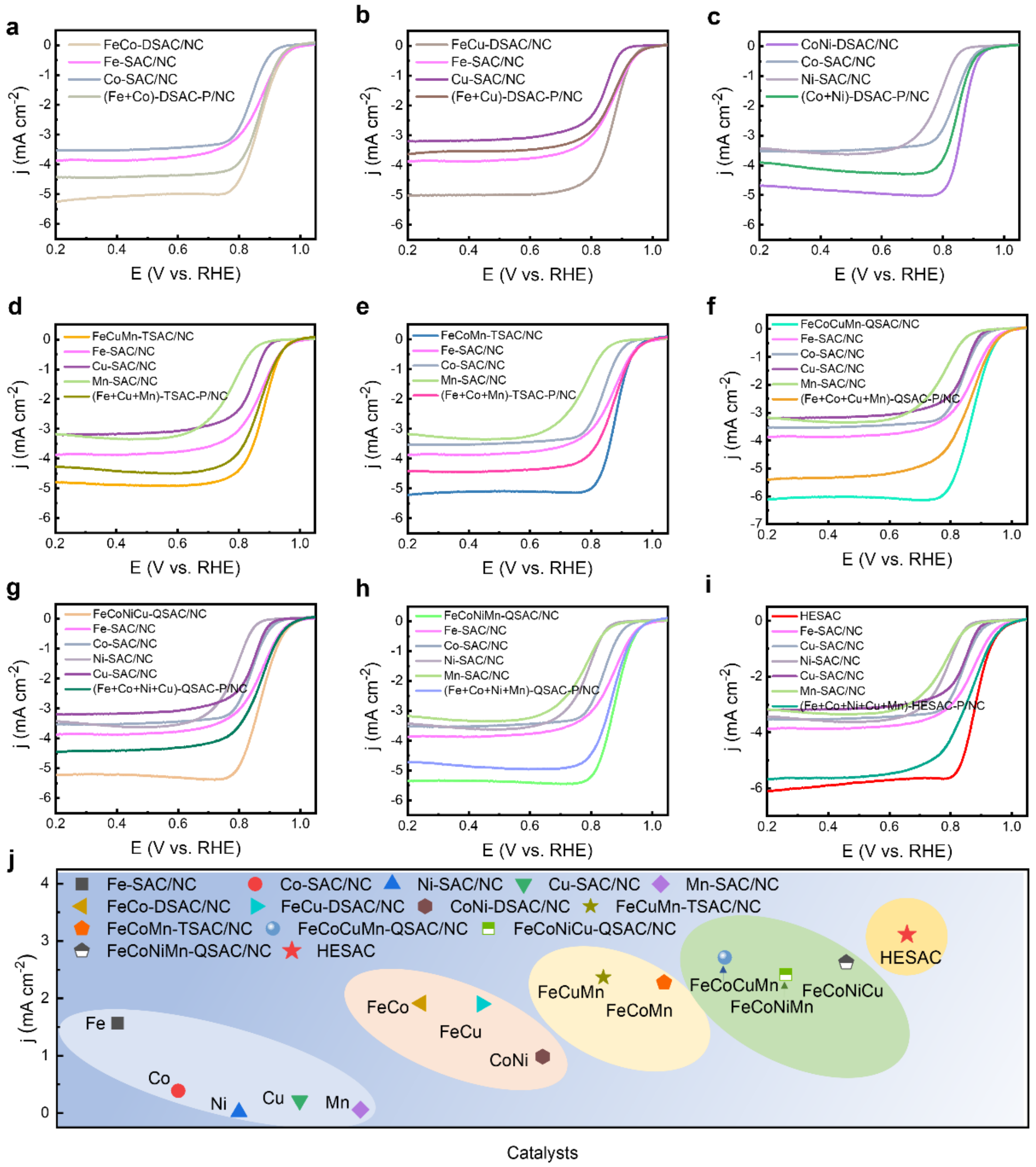

Supplementary Fig. 12 ORR performance of the prepared catalysts. **(a-i)** LSV curves of the prepared catalysts, **(j)** summary of the j_k of the prepared catalysts.

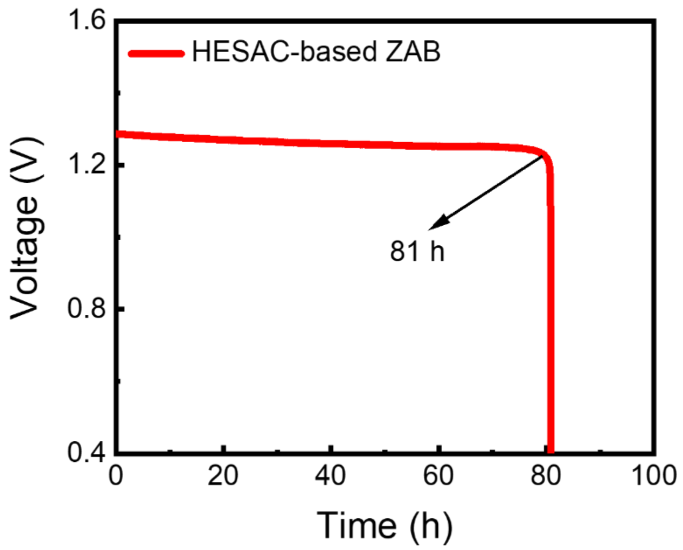

Supplementary Fig. 13 ZAB data. Discharge curve of the HESAC-based ZAB.

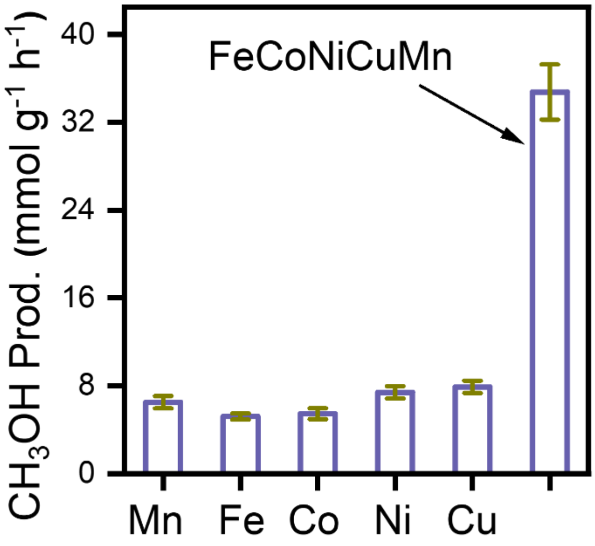

Supplementary Fig. 14 Catalytic performance of the direct conversion of CH_4. The yield of products of CH_3OH for Mn-SAC/NC, Fe-SAC/NC, Co-SAC/NC, Ni-SAC/NC, Cu-SAC/NC, and the quinary HESACs (FeCoNiCuMn).

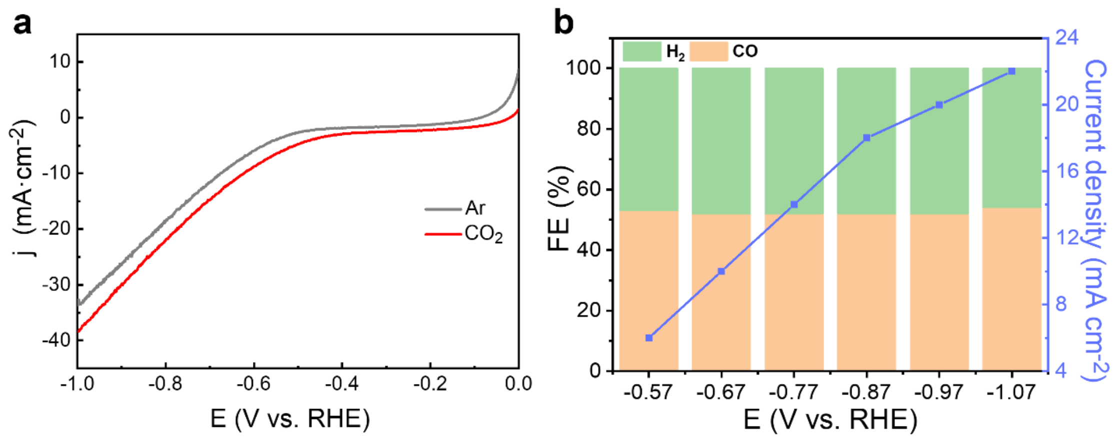

Supplementary Fig. 15 ECR performance of the prepread HESAC. **(a)** LSV curves of the quinary HESACs (FeCoNiCuMn) under Ar and CO_2, respectively. **(b)** FE of CO and H_2, and current density for quinary HESACs (FeCoNiCuMn).

Supplementary Table 1 Metal content of the quinary HEASCs (FeCoNiCuMn).

**Elements**	**wt. %**
Fe	0.39
Co	0.35
Ni	0.22
Cu	0.24
Mn	0.27
Total	1.47

Supplementary Table 2 Structural parameters extracted from the EXAFS fitting.

Sample	Scattering pair	CN	R(Å)	σ^2 (Å^2)	ΔE_0 (eV)	*R* factor
**HESAC-Fe**	Fe-N	3.8±0.9	2.00±0.05	0.007±0.005	-2.11	0.007
**HESAC-Co**	Co-N	4.1±1.3	1.96±0.05	0.015±0.005	-5.42	0.010
**HESAC-Ni**	Ni-N	3.9±0.3	1.85±0.02	0.013±0.009	-1.8	0.005
**HESAC-Cu**	Cu-N	3.9±0.4	1.94±0.06	0.008±0.01	6.42	0.002
**HESAC-Mn**	Mn-N	3.6±0.3	1.84±0.01	0.004±0.001	-1.20	0.001
	Mn-N-C	5.4±0.6	2.93±0.01	0.006±0.001	7.10	

CN is coordination number, R is the distance between absorber and backscatter atoms, σ^2 is Debye-Waller factor to account for both thermal and structural disorders, ΔE_0 is inner potential correction, *R* factor indicates the goodness of the fit.

Supplementary Table 3 ORR data of the prepared catalysts and Pt/C.

	E_{onset} (V vs. RHE)	$E_{1/2}$ (V vs. RHE)	j_L@ 0.2 V vs. RHE (mA cm^{-2})	j_k@ 0.90 V vs. RHE (mA cm^{-2})
**HESAC**	0.999	0.887	6.114	3.114
**NC**	0.915	0.729	4.560	0.139
**Pt/C**	0.982	0.866	5.494	1.963

Supplementary Table 4 Comparation of ORR performance of the quinary HESACs (FeCoNiCuMn) and Pt/C before and after ADTs.

	**Before ADTs**		**After ADTs**	
	**$E_{1/2}$ (V vs. RHE)**	**j_k@ 0.90 V vs. RHE ($mA\ cm^{-2}$)**	**$E_{1/2}$ (V vs. RHE)**	**j_k@ 0.90 V vs. RHE ($mA\ cm^{-2}$)**
**HESAC**	0.887	3.114	0.882	3.011
**Pt/C**	0.866	1.963	0.844	0.951

Supplementary Table 5 Comparison of the ORR performance of the prepared HESAC and recent reported SACs.

Samples	E_{ONSET} V vs. RHE	$E_{1/2}$ V vs. RHE	References
**HESAC**	**0.999**	**0.887**	**This work**
**Pt/C**	**0.982**	**0.866**	**This work**
Co SA/N-CNS-900	1.00	0.877	*J. Energy Chem.,* **2022**, 68, 184-194
Cu/Zn-NC	0.98	0.83	*Angew. Chem. Int. Ed.,* **2021**, 60 (25) 14005-14012
FeN_4-PN	1.00*	0.91	ACS Catal., **2021**, 11, 6304-6315
SA-CuNC	0.99*	0.78	*Adv. Energy Mater.,* **2021**, 11, 2100303
Fe SA-NSC-900	0.94	0.86	*ACS Energy Lett.,* **2021**, 6, 379-386
Mg-N-C	1.03	0.91	*Nat. Commun.,* **2020**, 11, 938
CoSAs-NGST	0.99	0.89	*Adv. Funct. Mater.,* **2021**, 31, 2010472
Co-CTF/KB	0.89	0.83	*Sci. China Mater.,* **2021**, 64: 2221-2229
Co-pyridinic N-C	0.99	0.87	*Adv. Energy Mater.,* **2020**, 2002592
Co-N-C	0.97	0.86	*Adv. Energy Mater.,* **2020**, 2002592
Fe-SA-NC	0.97*	0.86	*Nat. Commun*., **2020**, 11, 5892
Pt-SCFP/C-12	0.90	0.81	*Adv. Energy Mater.*, **2020**, 10 1903271.
Co-SAs/NSC	0.95	0.86	*J. Am. Chem. Soc.,* **2019**, 141, 20118-20126.
Co-SAs@NC	0.96	0.82	*Angew. Chem. Int. Ed.,* **2019**, 58, 5359-5364
Co-Nx-C	0.90*	0.83	*Adv. Mater.,* **2019**, 31, 1900592
SCoNC	1.00*	0.91	*Adv. Energy Mater.,* **2019**, 1900149
Fe-NCNWs	0.99	0.90	*ACS Catal.,* **2019**, 9, 5929-5934
Zn/CoN-C	1.004	0.861	*Angew. Chem. Int. Ed.,* **2019**, 131, 2648-2652
Co-SAs@NC	0.96	0.82	*Angew. Chem. Int. Ed.,* **2019**, 58 5359-5364

Fe-SAs/NSC	1.00	0.87	*J. Am. Chem. Soc.,* **2019**, 141, 51, 20118-20126
Cu-N_4-C	0.915	0.84	*ACS Nano,* **2019,** *13* (3), 3177-3187
FePc/Ti_3C_2Tx	0.93	0.88	*Adv. Mater.,* **2018**, 30, e1803220
Fe-ISA/SNC	0.98	0.89	*Adv. Mater.,* **2018**, 30, e1800588
Co SAs/N-C(900)	0.982	0.881	*Angew. Chem. Int. Ed.,* **2016**, 128, 10958-10963

* The data is extracted from LSV curves.

Supplementary Table 6 Comparison of the ORR performance of the prepared catalysts.

Catalysts	E_{onset} V vs. RHE	$E_{1/2}$ V vs. RHE	J_k @ 0.9 V mA cm^{-2}
Fe-SAC/NC	0.979	0.863	1.568
Co-SAC/NC	0.929	0.842	0.389
Ni-SAC/NC	0.871	0.784	0.020
Cu-SAC/NC	0.913	0.84	0.216
Mn-SAC/NC	0.885	0.772	0.059
FeCo-DSAC/NC	0.98	0.868	1.915
FeCu-DSAC/NC	0.967	0.871	1.903
CoNi-DSAC/NC	0.949	0.865	0.985
FeCuMn-TSAC/NC	0.967	0.877	2.365
FeCoMn-TSAC/NC	0.969	0.88	2.277
FeCoCuMn-QSAC/NC	0.985	0.872	2.714
FeCoNiCu-QSAC/NC	0.989	0.87	2.416
FeCoNiMn-QSAC/NC	0.975	0.877	2.627
HESAC	0.999	0.887	3.114
(Fe+Co)-DSAC/NC	0.965	0.869	0.169
(Fe+Cu)-DSAC/NC	0.977	0.867	1.388
(Co+Ni)-DSAC/NC	0.945	0.85	0.551
(Fe+Cu+Mn)-TSAC/NC	0.973	0.865	1.688
(Fe+Co+Mn)-TSAC/NC	0.979	0.87	1.865
(Fe+Co+Cu+Mn)-QSAC/NC	0.987	0.857	2.018

(Fe+Co+Ni+Cu)-QSAC/NC	0.983	0.867	1.785
(Fe+Co+Ni+Mn)-QSAC/NC	0.977	0.87	2.006
(Fe+Co+Ni+Cu+Mn)-HESAC/NC	0.994	0.858	2.216
